# Long‐term NAD+ supplementation prevents the progression of age‐related hearing loss in mice

**DOI:** 10.1111/acel.13909

**Published:** 2023-07-03

**Authors:** Mustafa N. Okur, Burcin Duan Sahbaz, Risako Kimura, Uri Manor, Jaimin Patel, Jae‐Hyeon Park, Leo Andrade, Chandrakala Puligilla, Deborah L. Croteau, Vilhelm A. Bohr

**Affiliations:** ^1^ Section on DNA Repair, National Institute on Aging National Institutes of Health Baltimore Maryland USA; ^2^ Waitt Advanced Biophotonics Center Salk Institute for Biological Studies La Jolla California USA; ^3^ Computational Biology & Genomics Core, National Institute on Aging National Institutes of Health Baltimore Maryland USA; ^4^ Danish Center for Healthy Aging University of Copenhagen Copenhagen N Denmark

**Keywords:** age‐related hearing loss, NAD+, nicotinamide riboside

## Abstract

Age‐related hearing loss (ARHL) is the most common sensory disability associated with human aging. Yet, there are no approved measures for preventing or treating this debilitating condition. With its slow progression, continuous and safe approaches are critical for ARHL treatment. Nicotinamide Riboside (NR), a NAD+ precursor, is well tolerated even for long‐term use and is already shown effective in various disease models including Alzheimer's and Parkinson's disease. It has also been beneficial against noise‐induced hearing loss and in hearing loss associated with premature aging. However, its beneficial impact on ARHL is not known. Using two different wild‐type mouse strains, we show that long‐term NR administration prevents the progression of ARHL. Through transcriptomic and biochemical analysis, we find that NR administration restores age‐associated reduction in cochlear NAD+ levels, upregulates biological pathways associated with synaptic transmission and PPAR signaling, and reduces the number of orphan ribbon synapses between afferent auditory neurons and inner hair cells. We also find that NR targets a novel pathway of lipid droplets in the cochlea by inducing the expression of CIDEC and PLIN1 proteins that are downstream of PPAR signaling and are key for lipid droplet growth. Taken together, our results demonstrate the therapeutic potential of NR treatment for ARHL and provide novel insights into its mechanism of action.

## Graphical abstract

Age‐related hearing loss (ARHL) is a common sensory disability associated with aging. Currently, no approved measures exist for preventing or treating this condition. Nicotinamide riboside (NR), an NAD+ precursor, has shown effectiveness in various diseases as well as noise‐induced and premature aging‐related hearing loss. Using two different wild‐type mouse strains, we show that long‐term NR administration prevents ARHL progression, particularly at high‐frequencies. Mechanistically, NR enhances synaptic transmission between auditory neurons and hair cells and targets a novel lipid droplet pathway in the cochlea by inducing the expression of CIDEC and PLIN1 proteins. Overall, our findings highlight NR's therapeutic potential for ARHL and provide insights into its mechanism of action.

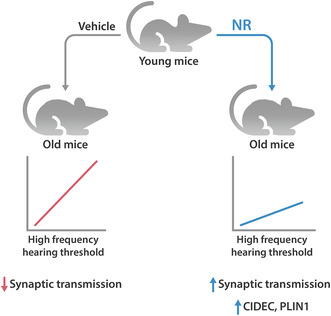

## 1 INTRODUCTION

Age‐related hearing loss (ARHL) is the most common sensory disability affecting the elderly human population. It manifests as a progressive, bilateral decline in hearing function starting with high‐frequency sounds. One in three adults over the age of 65 show clinically diagnosable hearing loss, and the risk doubles with every decade of life (Briggs, 2019). Due to its slow progression, ARHL is often overlooked. Yet, it is extensively associated with cognitive decline, social isolation, and accident risk. Combined with the projected increase in the global elderly population, ARHL poses an enormous public health challenge.

Despite its rising prevalence and medical cost, the etiology of ARHL is not clear. Age‐dependent changes in DNA damage accumulation, oxidative stress, mitochondrial dysfunction, and senescent‐associated inflammation are postulated underlying biological mechanisms leading to ARHL (Okur & Djalilian, 2022; Wang & Puel, 2020). Interestingly, the abundance of intracellular NAD+ levels plays a prominent role in correcting multiple aspects of the abovementioned biological events or ameliorating their cytotoxic outcomes (Fang et al., 2019; Okur, Fang, et al., 2020). NAD+ is a critical cofactor for numerous enzymes central to metabolism, longevity, and neuroprotection (Verdin, 2015). Notably, cochlear NAD+ levels decline in response to noise exposure, and NAD+ augmentation restores noise‐induced neurite retraction from inner hair cells (IHCs), suggesting that NAD+ levels are critical for proper cochlear function (Brown et al., 2014). Indeed, we previously showed that NAD+ supplementation improves synaptic connectivity in the cochlea and prevents the progression of hearing loss in a premature aging disease model associated with dramatic hearing loss (Okur, Mao, et al., 2020). However, the impact of long‐term NAD+ supplementation on ARHL has not been tested using a direct NAD+ precursor.

Nicotinamide riboside (NR) is a NAD+ precursor found in foods including fruits, vegetables, meat, and milk (Yoshino et al., 2018). NR is well tolerated in rodents and humans and its oral intake is shown to boost NAD+ levels in a variety of tissues and organs including the brain and heart (Diguet et al., 2018; Martens et al., 2018). Using two different mouse models, we show that long‐term NR administration reduces the progression of ARHL, particularly of high‐frequency sounds. Mechanistically, we find that oral NR administration restores age‐associated NAD+ decline in mouse cochlea. Also, our cochlear transcriptomic analysis demonstrates that NR upregulates pathways associated with synaptic connectivity. Our in‐depth electrophysiological and histological analyses in cochlea support these results and show that NR enhances synaptic connectivity between cochlear sensory cells and afferent primary neurons in mice. In addition, we find that NR administration elevates the expression of key proteins involved in lipid droplet growth such as CIDEC and PLIN1, illustrating a novel pathway targeted by NR. Overall, these findings demonstrate the translational potential of NR on ARHL and provide novel insights into its mechanism of action.

## 2 RESULTS

### 2.1 NR prevents the progression of ARHL

**FIGURE 1:**
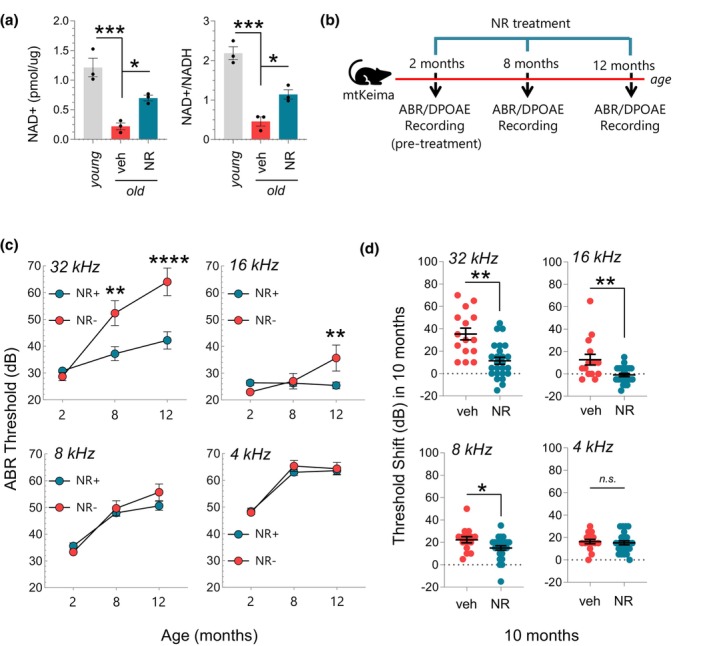
NAD+ supplementation using NR prevents the progression of age‐related hearing loss in WT mice (mtKeima). (a) The total NAD+ levels per ug of the cochlea (left panel) and relative NAD+/NADH levels (right panel) were measured in the cochlea of young (2‐month‐old), old (12‐month‐old), and NR‐treated old mice (12‐month‐old). *N* = 3 and ordinary one‐way ANOVA were used to determine significant differences. (b) Outline for NR treatment and ABR/DPOAE recordings in WT mice (mtKeima). (c) ABR thresholds for WT and NR‐treated WT mice at 2, 8, and 12 m of age. A total of 40 WT mice were tested for hearing capacity at the age of 2 m and then randomly split into two groups of NR‐treated (*N* = 25) and non‐treated (*N* = 15). NR treatment started at the age of 2 m. ABRs in both groups were measured again at the age of 8 and 12 m, which correspond to 6 and 10 m of NR treatment respectively. Mixed effect analysis with Sidak's multiple comparison test was used to determine significant differences. (d) Threshold shifts at 8, 16, and 32 kHz in NR‐treated and untreated groups. ABR data in (c) were used to calculate the hearing shift. Each dot represents a data point for an individual mouse. Two‐tailed *t*‐test was used to determine significant differences. Mean ± SE, *p ≤ 0.05, **p ≤ 0.01, ***p ≤ 0.001, ****p ≤ 0.0001 and n.s., not significant.

NAD+ levels decline upon aging in various rodent tissues including the kidney, brain, heart, and lung (Braidy et al., 2011). To assess whether the same phenomenon applies to cochlear tissue, we compared cellular NAD+ levels in the cochlea of young (2‐month‐old) and old mice (12‐month‐old) and tested if oral NR administration restores NAD+ levels. We found that total NAD+ and relative NAD+/NADH levels were indeed lower in the aged cochlea, and this decline was rescued significantly with NR administration (Figure 1a). Given that NAD+ levels are associated with cochlear function (Brown et al., 2014), we next tested the effect of long‐term NR administration on hearing loss in aged mice (Figure 1b). One of the primary functions of NAD+ is to improve mitochondrial health by inducing mitochondrial turnover (Fang et al., 2019; Okur, Fang, et al., 2020). To gain insight into the mechanism of action of NAD+'s benefit on hearing loss, we used WT (mtKeima) mice with a reporter gene to also assess mitochondrial degradation (mitophagy) in the cochlea (Malide et al., 2016) (see Supplementary Methods for details). We used the Auditory Brain Response (ABR) system to measure hearing capacity. ABR measures brain wave activity in response to sound stimuli at different frequencies (Hz) and decibel (dB) intensities (see Supplementary Methods for details). We observed an age‐dependent elevation in hearing loss in the non‐treated group at all frequencies tested, showing that the WT mouse strain used in these experiments suffers from ARHL (Figure 1c). Remarkably, NR administration prevented the progression of ARHL specifically at high frequencies while no effect at lower frequencies was observed (Figure 1c). This phenomenon was observed in both males and females (Figure S1) with similar differences. We next compared the hearing threshold shift in treated and non‐treated groups to analyze NR's effect on hearing loss in individual mice. We found that NR not only prevented hearing loss progression but also improved high‐frequency hearing in a subset of mice in the NR‐treated group (Figure 1d).

### 2.2 NR elevates wave I and III ABR amplitudes

**FIGURE 2:**
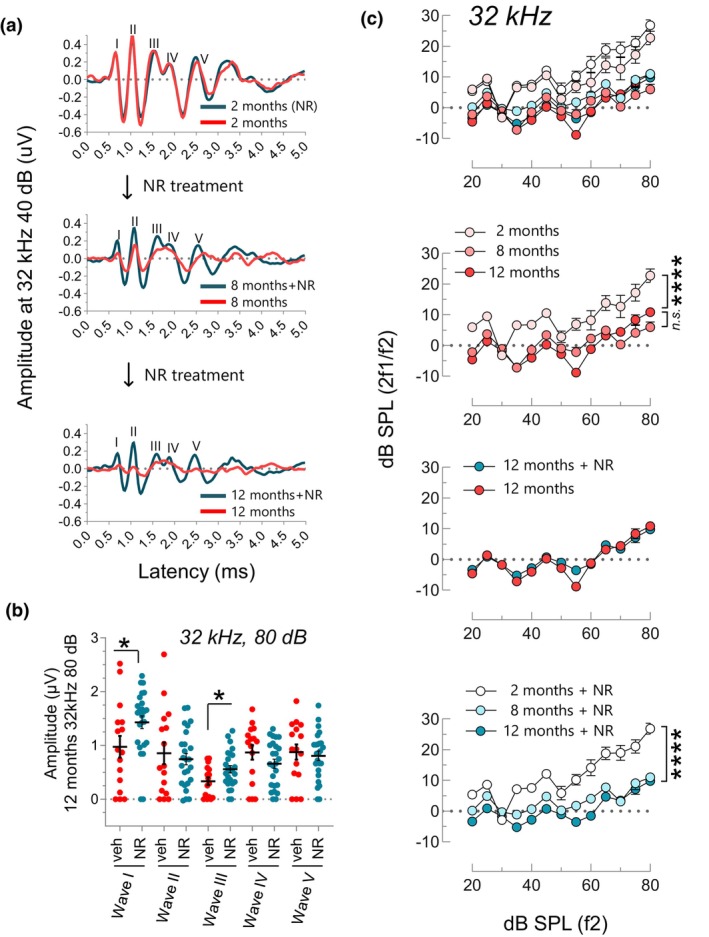
NR does not affect DPOAE but elevates wave I and III ABR amplitudes in WT mice (mtKeima). (a) Averaged ABR waveforms in NR‐treated and non‐treated mice resulting from a 32 kHz 40 dB SPL stimulus presented at 2, 8, and 12 m of age. (b) Quantification of wave I‐V amplitudes at 12 m. NR treatment significantly preserves wave I and III amplitudes. Each dot represents a data point for an individual mouse. Two‐tailed *t*‐test was used to determine significant differences. (c) DPOAE levels at 32 kHz are shown at the age of 2, 8, and 12 m of age in NR‐treated (*N* = 25) and non‐treated mice (*N* = 13). The area under the curve (above −10 on the *y*‐axis) is calculated for each sample and two‐way ANOVA with Tukey's post hoc test was used for statistical analysis. Mean ± SE. *p ≤ 0.05, ****p ≤ 0.0001 and n.s., not significant.

ABRs are electrical potentials and recorded as five to seven waves in the first 10 ms due to synchronous firing of nerve fibers after an auditory stimulus. The first wave (wave I) captures the activity of IHCs, afferent auditory neurons, and the synaptic connectivity between them. The following waves (waves II, III, IV, and V) are believed to correspond to locations descending through the auditory pathway corresponding to the cochlear nucleus, superior olivary complex, lateral lemniscus, and inferior colliculus, respectively. The reduction in the wave magnitude or increase in wave latency indicates a hearing deficit while the impacted wave provides valuable information for locating the deficit along the auditory pathway. We reconstructed average ABR waveforms and further examined the wave activity to gain more insight into NR's benefit on hearing (Figure 2a). We found that the average wave amplitude (40 dB at 32 kHz) declines as a function of age in both treated and non‐treated groups (Figure 2a, compare the top and bottom panels). However, despite the decline, the amplitude of the waveforms in the NR‐treated group was prominently higher than the ones in the non‐treated group at 12 months (m) of age although they were similar at young ages (Figure 2a, bottom panel). We next quantified and compared the amplitudes of individual waves in each mouse (Figure 2b). Wave amplitudes fade away at lower decibel sounds so we analyzed wave amplitudes in response to higher decibel sound (80 dB) to account for all mice including the ones with prominent hearing loss. We found that NR specifically enhanced Wave I and Wave III amplitudes in the treated vs. non‐treated group (Figure 2b). These results indicate NR enhances cell function along the auditory pathway. This could include either or both sensory and auditory nerve cells and synapses thereof in the cochlea (Wave I), and nerve cells in the superior olivary complex in the brainstem (Wave III).

### 2.3 NR does not affect outer hair cell function

Due to their vulnerable nature, outer hair cells (OHCs) are lost early during aging, prominently contributing to ARHL. Thus, we also tested if NR benefits hearing by preventing age‐associated loss of OHCs. To address this, we measured DPOAE levels, which is an overall indicator of OHC functionality (see Supplementary Methods for details). We observed an age‐associated decline in DPOAE levels in the non‐treated group at 32 kHz while no significant difference was observed at lower frequencies (Figure 2c and Figure S2). Interestingly, NR treatment did not impact overall DPOAE levels, suggesting that NR's benefit on ARHL involves a mechanism other than its effect on OHCs (Figure 2c, the third panel from the top).

### 2.4 NR upregulates biological events associated with synaptic transmission and the PPAR signaling pathway

**FIGURE 3:**
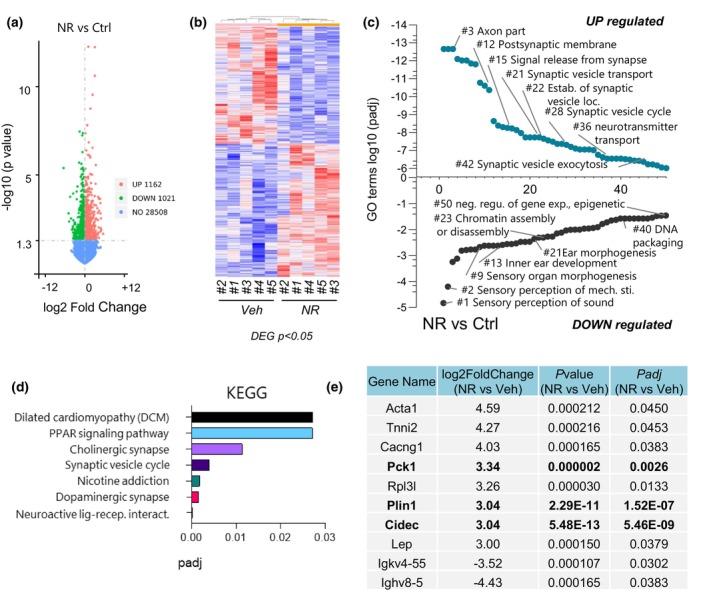
The transcriptomic analysis in NR‐treated and non‐treated cochlea in WT mice (mtKeima) (*N* = 5, 12‐month‐old). (a) The number of up‐ and down‐regulated genes with a *p*‐value ≤0.05 in NR‐treated and non‐treated mice cochlea. (b) Heatmap showing clustering of differentially expressed genes (DEG) (*p* ≤ 0.05) in NR‐treated and non‐treated mice cochlea. (c) Graph showing the top 50 up‐ or down‐regulated GO terms from the WT cochlea ±NR treatment. A *p*adj‐value ≤0.05 were the cutoff used for significance. (d) Graph showing the up‐regulated KEGG pathways from the WT cochlea ±NR treatment. A *p*adj‐value ≤0.05 were the cutoff used for significance. No significant down‐regulated KEGG pathways were detected. (e) The table shows a list of the top 10 genes with the highest value of fold change (up‐ or down‐regulated). A *p*adj‐value ≤0.05 and fold‐change (log2) ≥3 were the cut‐offs used for significance.

**FIGURE 4:**
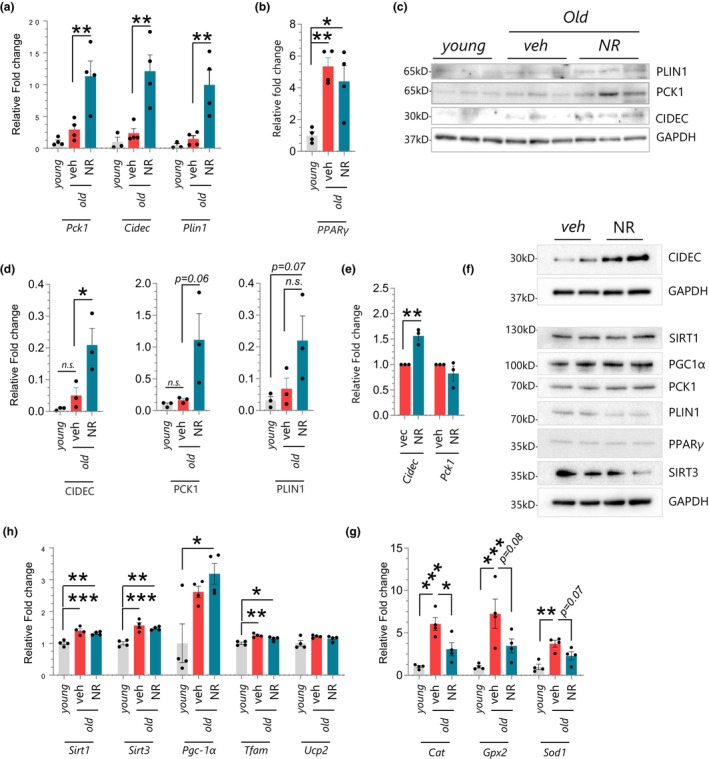
NAD+ supplementation using NR modulates the expression of CIDEC and PLIN1, key proteins of lipid droplets dynamics in WT mice (mtKeima). (a, b) Quantitative RT‐PCR results demonstrate the relative fold change in Pck1, Cidec, Plin1, and PPARγ in the cochlea of young (2‐month‐old), old (12‐month‐old), and NR‐treated old mice (12‐month‐old). (*N* = 4 mice per group, one‐way ANOVA with Tukey's post hoc test was used for statistical analysis.) (c) Western blot depicts protein expression in the cochlea of young (2‐month‐old), old (12‐month‐old), and NR‐treated old mice (12‐month‐old). (d) The graph demonstrates the quantification of the average signal from the Western Blot in Figure 4c using the NIH ImageJ program. One‐way ANOVA with Tukey's post hoc test was used for statistical analysis. (e) Quantitative RT‐PCR results demonstrate the relative fold change in Pck1 and Cidec following NR treatment (1 mM, 24H) in HEI‐OC1 cells. Each dot represents a data point from independent biological repeats. (f) Western blot depicts protein expression from HEI‐OC1 cells following NR treatment (1 mM, 24H) from two independent biological repeats (lanes 1 and 3 demonstrate experiment #1, lanes 2 and 4 demonstrate Experiment #2). (g, h) Quantitative RT‐PCR results demonstrate the relative fold change in genes in the cochlea of young (2‐month‐old), old (12‐month‐old), and NR‐treated old mice (12‐month‐old). (*N* = 4 mice per group, one‐way ANOVA with Tukey's post hoc test was used for statistical analysis.) Mean ± SE. *p ≤ 0.05, **p ≤ 0.01, ***p ≤ 0.001, and n.s., not significant.

To investigate the biological mechanism of NR action on ARHL, we used unbiased RNA sequencing (RNA‐seq) to identify the transcriptomic profiles of the cochlea from treated and non‐treated groups. We found 1162 up‐regulated genes and 1021 down‐regulated genes with NR treatment (Figure 3a). When hierarchical clustering was performed on those differentially expressed genes, NR‐treated samples cluster more closely together, suggesting that NR led to a similar gene expression pattern in the cochlea in treated mice (Figure 3b). We next performed gene ontology (GO) analysis to identify biological processes that are altered with NR treatment. Remarkably, NR treatment up‐regulated many terms associated with synaptic transmission such as postsynaptic membrane, signal release from the synapse, synaptic vesicle transport, and synaptic vesicle cycling (Figure 3c), consistent with the wave‐form analysis in Figure 2, as well as with our previous study (Okur, Mao, et al., 2020). Down‐regulated GO terms, on the other hand, included sensory perception, inner ear development, and ear development and morphogenesis. Notably, mitochondria‐related terms were not among the most significantly changed GO‐term list (Figure 3c). This was surprising given the role of NAD+ in mitochondrial homeostasis. We extended our analysis and performed KEGG enrichment to identify associated biological pathways with treatment. We found that seven pathways were significantly up‐regulated with treatment while no down‐regulated pathways were observed (Figure 3d). Interestingly, ‘synaptic vesicle cycle’ was an upregulated term in KEGG as it was in the GO analysis (Figure 3c,d), supporting the notion that NR treatment promotes biological pathways associated with synaptic transmission. Among the upregulated list of KEGG pathways, we also identified that NR improved PPAR signaling, a family of transcription factors that regulate metabolic homeostasis (Grygiel‐Górniak, 2014). Recent studies showed that PPAR activation protects the cochlea from oxidative stress, pointing out a potential biological pathway that NR targets (Sekulic‐Jablanovic et al., 2017). PPAR regulates lipid metabolism and promotes the formation of lipid droplets, which are cellular organelles that store, release, and process lipids and proteins (Gorga et al., 2017). Remarkably, in the list of top genes that are most up‐ or down‐regulated with treatment (Figure 3e), we identified various genes such as *Cidec*, *Plin1*, *and Pck1* that are targeted by the PPARγ transcription factor and play key roles in lipid droplet formation. PLIN1 interacts with and activates CIDEC (FSP27) to regulate lipid droplet enlargement in mouse 3T3‐L1 preadipocytes and human adipocytes (Grahn et al., 2013; Sun et al., 2013). PCK1 is a metabolic enzyme that has a role in lipogenesis (Xu et al., 2020). Although predominantly found in adipocytes, lipid droplets are present in most cells. Recent studies postulate that lipid droplets protect the delicate structure of the cochlea (Urrutia & Kalinec, 2015). We, therefore, focused on CIDEC, PLIN1, PCK1, and PPARγ, validating their gene expression levels using RT‐PCR. We also compared their levels to those in the young cochlea to determine changes with aging. Despite no significant effect of aging, the gene expression levels of *Cidec*, *Plin1*, and *Pck1* increased dramatically with NR treatment (Figure 4a). These results were consistent with the gene expression patterns in RNA‐Seq, confirming the reliability of RNA‐seq results in this study. PPARγ levels, on the other hand, increased upon aging and showed no difference after NR treatment (Figure 4b). We observed a similar trend in protein expression levels of these genes and found that NR significantly increases CIDEC levels while there was a trend towards elevated PLIN1 and PCK1 levels (Figure 4c,d). Additionally, we used cultured mouse cochlear cell lines (HEI‐OC1) to assess NR's effect more directly. We found that only CIDEC's levels were elevated with NR treatment whereas there was no change in PCK1 levels and a slight reduction in PLIN1 levels in HEI‐OC1 cells (Figure 4e,f). These results emphasized that NR consistently upregulates CIDEC expression both in cochlear tissues and in HEI‐OC1 cells.

Given the role of NAD+ in mitochondrial homeostasis, we also examined the expression levels of mitochondria‐related genes (*Sirt1*, *Sirt3*, *Pgc‐1α*, *Tfam*, *Ucp2*) in the cochlea and found an age‐related elevation in their levels, with the exception of *Ucp2*. When treated and non‐treated old cochlea were compared, no significant impact of NR was observed on mitochondria‐related genes, which was in accord with RNAseq results in which no mitochondrial terms were identified in the top GO‐term list from the cochlear samples (Figure 4g). We observed similar results in cultured cochlear cells in which there was no change in the expression of Sir1, Sirt3, Pgc‐1α, and PPARγ with NR treatment (Figure 4f and Figure S3).

The mtKeima strain was designed for ex‐vivo imaging of mitochondria and mitophagy events (Malide et al., 2016). It expresses a pH‐dependent mtKeima protein and is resistant to lysosomal proteases. When mitochondria are in neutral pH, they fluoresce green but when in lysosomes, as during mitophagy, they are magenta. Thus, this strain permits the analysis of mitochondrial pools in and out of lysosomes. In our ex‐vivo studies on live cochlear tissue, we show that NR treatment did not have a significant impact on mitophagy in auditory neurons in the base turn of the cochlea (Figure S4). These results suggest that NR improves cochlear function with limited direct impact on mitochondria. However, lipid droplets are known to interact with mitochondria and reduce excessive mitochondrial reactive oxygen species overflow and fatty acid oxidation, contributing to the antioxidant capacity of cells (Aon et al., 2014; Bailey et al., 2015). Indeed, we observed that NR leads to a significant reduction in catalase (*Cat*), while there was a trend for a reduction in the levels of oxidative stress‐related genes glutathione peroxidase 1 and superoxide dismutase 1 (*Gpx*, and *Sod1*) that were elevated in the cochlea of old mice (Figure 4h), suggesting an indirect effect of NR on mitochondrial homeostasis and function.

Taken together, our results suggest a novel pathway of NR acting along the PPARγ‐CIDEC, ‐PLIN1, ‐PCK1 axis, and that it potentially contributes to lipid droplet formation and protection of the cochlear cells from cytotoxic damage.

### 2.5 NR delays hearing loss progression in aged female mice at 24 kHz frequency

**FIGURE 5:**
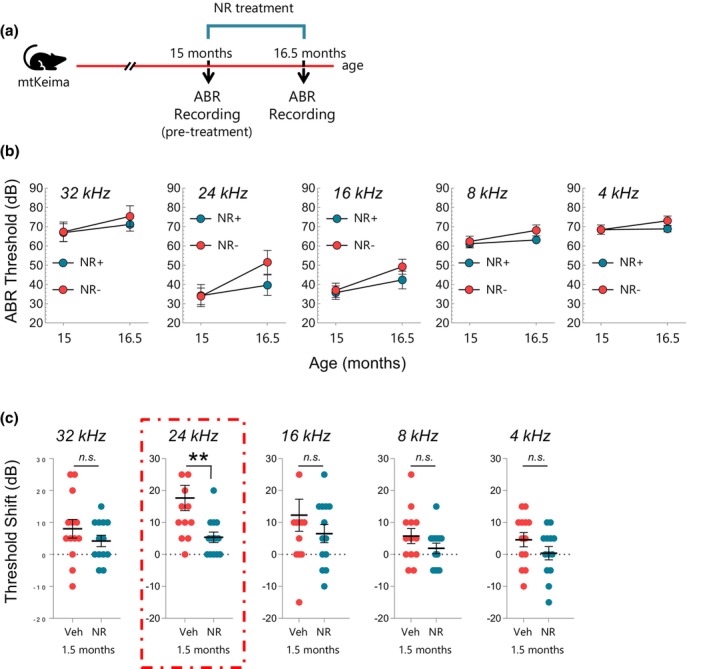
NR delays hearing loss progression in aged female WT mice (mtKeima) at 24 kHz frequency. (a) Outline for NR treatment and ABR recordings in WT mice (mtKeima). (b) ABR thresholds for NR‐treated and non‐treated mice at 15, and 16.5 m of age. A total of 26 mice were tested for hearing capacity at the age of 15 m and then split into two groups of NR‐treated (*N* = 13; 6 M, 9F) and non‐treated (*N* = 13; 6 M, 9F). Groups are gender‐matched and hearing capacity‐matched at 32 kHz at the age of 15 m. NR treatment started at the age of 15 m. ABRs in both groups were measured again at the age of 16.5 m, which corresponds to 1.5 m of NR treatment. Mixed effect analysis with Sidak's multiple comparison test was used to determine significant differences. (c) NR treatment reduces the increased threshold shift at 24 kHz. ABR data in (b) were used to calculate the hearing shift. Two‐tailed *t*‐test was used to determine significant differences. Each dot represents a data point for an individual mouse. Mean ± SE. **p ≤ 0.01, and n.s., not significant.

We next tested if NR treatment was still beneficial for hearing loss after a hearing deficit had already developed. To address this, we first confirmed the substantial hearing loss in mtKeima mice at 15 m of age and then administered NR for 1.5 m (Figure 5a,b). We found that NR had no significant impact on hearing thresholds at any given frequency (Figure 5b), possibly due to a slight increase in ABR thresholds limiting the room for improvement. However, when individual threshold shifts were investigated, we found that NR treatment reduced threshold shifts significantly at 24 kHz, which only impacted females (Figure 5c and Figure S5). These results show that NR treatment at a late stage may still be beneficial, at certain frequencies and gender, even after hearing loss has already formed.

### 2.6 Long‐term NR administration reduces the progression of ARHL in WT mice with natural hearing loss

**FIGURE 6:**
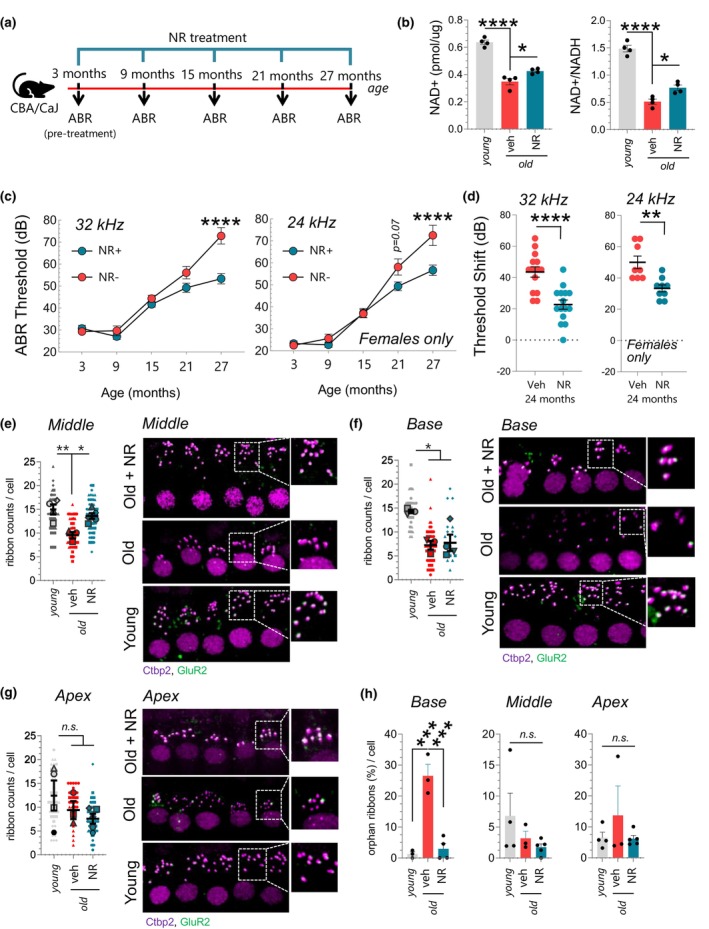
NAD+ supplementation using NR reduces the progression of age‐related hearing loss in WT mice (CBA/CaJ). (a) Outline for NR treatment and ABR recordings in WT mice (CBA/CaJ). (b) The total NAD+ levels per ug of the cochlea (left panel) and relative NAD+/NADH levels (right panel) were measured in the cochlea of young (3‐month‐old), old (27‐month‐old), and NR‐treated old mice (27‐month‐old). *N* = 4. (c) ABR thresholds for WT and NR‐treated WT mice at 3, 9, 15, 21, and 27 m of age. A total of 29 WT mice were tested at the age of 3 m and then randomly split into two groups of NR‐treated (*N* = 15) and non‐treated (*N* = 14). NR treatment started at the age of 3 m. ABRs in both groups were measured again at the age of 9, 15, 21, and 27 m, which correspond to 6, 12, 18, and 24 m of NR treatment respectively. (d) NR treatment reduces the increased threshold shift at 24 and 32 kHz. ABR data in (c) were used to calculate the hearing shift. The left panel in (e–g) demonstrates the average synaptic ribbon count per IHC while the right panel shows a representative image of immunostaining for synaptic ribbons (violet, anti‐Ctbp2 (Ribeye) and post‐synaptic receptor (green, anti‐GluR2a) of cochlear middle, base, and apex segments. The anti‐Ctbp2 faintly stains hair cell nuclei. Zoom‐in images on the right‐top corners of the panels illustrate the juxtaposition of ribbon–receptor pairs in selected areas. (h) The graphs show the average number of orphan ribbons per inner cell. The violet puncta (anti‐Ctbp2) without green puncta (anti‐GluR2a) juxtaposition is considered an orphan ribbon. Mean ± SE. Mixed effect analysis with Sidak's multiple comparison test in (c), Two‐tailed *t*‐test in (d) and one‐way ANOVA with Tukey's post hoc test in (a and e–h) were used to determine significant differences. Each dot represents a data point for an individual mouse. Mean ± SE. *p ≤ 0.05, **p ≤ 0.01, ***p ≤ 0.001, ****p ≤ 0.0001 and n.s., not significant.

Our results so far show that NR administration reduces the progression of ARHL in mtKeima mice. To confirm these results, we also tested the impact of NR administration on ARHL using a different WT mouse strain (CBA/CaJ) that displays slower hearing loss progression than the mtKeima mouse strain and therefore better represents ARHL (Figure 6a). Indeed, this model is widely used in auditory research, particularly for ARHL‐related studies (Sha et al., 2008). In accord with our previous results in the mtKeima strain, CBA/CaJ also displayed a decrease in total NAD+ and NAD/NADH levels in the cochlea upon aging, which was partially restored with NR. We next investigated the underlying potential mechanisms leading to NAD+ decline in the cochlea and thus assessed potential genes involved in NAD+ synthesis/degradation. We observed a significant age‐related decline in *NMNAT2* and a trend of an increase in *CD157* levels (Figure S8). We did not observe any significant alterations in the levels of other genes involved in NAD+ synthesis (Figure S8) or the members of the Sirtuin or Parp family (data not shown).

We next tested whether NR‐mediated improvement in NAD+ levels reflected itself in hearing thresholds in CBA/CaJ mice (Figure 6b). We found that 24 m of NR administration reduces ARHL progression in CBA/CaJ mice at 32 kHz frequency in both genders (Figure 6c, left panel) while improvement at 24 kHz was observed only in females (Figure 6c, right panel). No significant difference was observed at lower frequencies in both genders (Figure S6). When hearing threshold shifts were compared, we found that the NR‐treated group developed less hearing loss per mouse compared to the non‐treated group at 32 kHz (both genders) and at 24 kHz (only females) (Figure 6d and Figure S7). These results also demonstrate NR's benefit on ARHL is not strain specific. Given the role of NR in synaptic connectivity (Figure 3c), we next investigated NR's effect on the integrity of synaptic transmission between sensory IHCs and auditory neurons. To address this, we analyzed synaptic ribbon counts in IHCs, which are electron‐dense structures that tether synaptic vesicles at the presynaptic active zones and facilitate continuous synaptic transmission (Kujawa & Liberman, 2015; Safieddine et al., 2012; Sergeyenko et al., 2013). The cochlea consists of base, middle, and apex regions. High‐frequency sound is sensed at the base of the cochlea whereas low frequencies are sensed at the apex. We observed an age‐associated decline in synaptic ribbon counts in the base and middle regions of the cochlea (Figure 6e–g, violet puncta), consistent with the decline in ABR levels at high‐frequency tones in the old mice (Figure 6c). Remarkably, we found that NR restored the reduction in ribbon counts in the middle region of the cochlea, potentially contributing to hearing in the mid‐frequency range (16–32 kHz; Figure 6e). However, no significant improvement was observed in the number of ribbon counts in the base region (Figure 6f). The apex region, on the other hand, displayed no significant change in synaptic ribbon counts upon aging or with NR treatment (Figure 6g). Given that NR improves hearing at 32 kHz (Figure 6c), the lack of effect on synaptic ribbons by NR in the base region was surprising. One potential mechanism could be that NR exerts its impact in this region by preserving hair cell innervation by afferent auditory neurons, leading to improved synaptic transmission. To gain insight into this mechanism, we examined the status of orphan ribbons that are unpaired ribbons with the post‐synaptic receptor, potentially affecting the rates of auditory neuron firing (Buran et al., 2010; Frank et al., 2010). We observed an age‐related increase in the number of orphan ribbons (violet only puncta without green juxtaposition in Figure 6e–g) specifically in the base region (Figure 6h). Interestingly, this increase in the number of orphan ribbons was fully reversed by NR treatment (Figure 6h, left panel). The apex and middle regions displayed no significant change in the number of orphan ribbon counts upon aging or with NR treatment (Figure 6h, middle and right panels).

## 3 DISCUSSION

ARHL is the most common type of sensorineural hearing loss. Current treatment approaches for ARHL have failed, including those targeting reactive oxygen species reduction. The slow progression of ARHL adds to the challenge for intervention because it requires treatment approaches safe for long‐term use. Our previous findings showed that NAD+ supplementation using NR effectively prevented the progression of hearing loss in premature aging models (Okur, Mao, et al., 2020). NR can be administered orally and has no known serious side effects, and many safety studies have been done (Martens et al., 2018), making it a good candidate for long‐term administration. Most notably, using two different WT mouse strains, we found that long‐term NR administration, for the duration of 1 or 2 years, partially prevents ARHL progression (Figures 1 and 6). Our studies also show that NR administration delays the further deterioration of already existing hearing loss in mice but only in females and at a specific frequency (24 kHz; Figure 5). This limited and mild effect of NR on established hearing loss might result from a slight increase in hearing thresholds, limiting the room for improvement by NR, and thus warrants further studies investigating the effect of long‐term NR administration on established hearing loss.

We investigated potential underlying biological mechanisms of NAD+ decline and repletion in the cochlea. We found that the transcript levels of *NMNAT2* and *CD157* altered during aging in the cochlea (Figure S8). NMNAT2 plays a role in the NAD+ salvage pathway and mediates NMN to NAD+ conversion while CD157 catalyzes NAD+ hydrolysis (Covarrubias et al., 2021). These results suggest that age‐dependent NAD+ decline in the cochlea is associated with the abnormalities in both NAD+ synthesis and degradation. To identify the role of NAD+ repletion on the cochlea, we first performed a cochlear transcriptomic analysis and found that several terms associated with synaptic transmission are up‐regulated by NR treatment (Figure 3c). In accordance with this, our in‐depth wave‐form analysis showed that NR prevents age‐associated reduction in Wave I amplitude (Figure 2b), which is indicative of the number of neurons firing between sensory and auditory nerve cells. Indeed, we observed that NR treatment rescues the age‐related decline in synaptic ribbon counts in the middle turn of the cochlea (Figure 6e). Synaptic ribbons are largely composed of RIBEYE (Ctbp2) proteins through multiple RIBEYE‐RIBEYE interactions (Magupalli et al., 2008). Interestingly, RIBEYE has NAD+/NADH binding pocket that modulates synaptic ribbon assembly and activity, suggesting a potential mechanism of action of NR. However, although NR improved high‐frequency hearing (Figure 6c), it did not increase the synaptic ribbon counts in the base region that corresponds to sensing high‐frequency tones (≥32 kHz) (Figure 6f). Therefore, we speculated that NR benefits hearing through additional mechanisms besides the upregulation of ribbon counts. Indeed, a previous study showed that NR prevents noise‐induced neurite retraction from IHCs (Brown et al., 2014). In line with these findings, we found that NR prevented an age‐related increase in the number of orphan ribbons specifically in the base region, suggesting that NR preserves the innervation of IHCs in this region. We propose that the increased number of orphan ribbons with age reflects a deterioration of afferent auditory neuron dendrites and that the reduction in orphan ribbons (and correspondingly improved Wave I amplitudes) with NR treatment reflects the preservation of afferent auditory neuron innervation and function. Given the decrease in orphan ribbons, but no change to hair cell ribbon counts in the base, we speculate that the main target of NR in this region is the auditory afferent neurons. Altogether, our results suggest that NR benefits hearing by contributing to synaptic stability between sensory cells and auditory primary neurons. However, we do not rule out the possibility that NR impacted other regions along the auditory pathway. In fact, we observed that NR also significantly improved Wave III amplitude, which represents the number of neurons firing in a superior olivary complex in the brainstem.

Besides the NR‐mediated upregulation of synaptic transmission, our transcriptomic analysis also indicates molecular signatures of lipid droplet regulation as a potential underlying mechanism of NR's benefit on hearing loss. Lipid droplets have long been considered lipid storage units of the cells, but it is now more apparent that their expansion and shrinkage are highly dynamic and tightly coupled to their interaction with other organelles to control energy homeostasis and cellular stress. For instance, lipid droplets accumulate free acids in their units and move them into mitochondria when needed for energy demand, reducing free acid in the cytosol and hence lipotoxicity while contributing to energy homeostasis (Olzmann & Carvalho, 2019). Here, we show for the first time that NAD+ supplementation using NR elevates the expression of key proteins of lipid droplet dynamics, CIDEC and PLIN1, in the cochlea. CIDEC protein binds to lipid droplets and regulates their enlargement (Grahn et al., 2013). PLIN1, a lipid droplet coating protein, binds to CIDEC and regulates lipid storage and lipolysis (Grahn et al., 2013; Hansen et al., 2017). PLIN1 directly interacts with Mfn2, a mitochondrial fusion protein, and facilitates the contact between mitochondria and lipid droplets, mediating lipolytic processes and cellular metabolism (Boutant et al., 2017). In accord with this, recent data showed that patients with the Mfn2 variant (D414V) exhibit a hearing loss phenotype (Sharma et al., 2021). NR‐mediated increases in CIDEC and PLIN1 hence suggest a potential regulation of lipid droplet dynamics by NAD+. However, the molecular basis of how NR modulates CIDEC and PLIN1 is not yet clear. *Cidec* and *Plin1* gene promoters contain functional PPAR‐responsive elements and are targeted by PPARγ (Shimizu et al., 2004). PPAR activation protects the cochlea from oxidative stress (Sekulic‐Jablanovic et al., 2017). We identified the PPAR signaling pathway in NR‐treated cochlea using RNA seq analysis (Figure 3d). Interestingly, it was shown that the inhibition of NAD+ synthesis lowers α‐ketoglutarate‐mediated PPAR expression in 3T3‐L1 cells (Okabe et al., 2020). However, we did not observe a significant change in the levels of PPARγ in NR‐treated cochlea or cochlear cells, suggesting that NR might modulate PPARγ activity or act downstream of the PPARγ pathway. These observations warrant further investigations to elucidate the effects of NR on lipid droplet dynamics and its impact on hearing loss.

Our results show that NR particularly prevents hearing loss at higher frequencies while its impact fades with decreasing frequency, suggesting that NR predominantly modulates the cochlear middle and base segments. These results may potentially reflect higher firing rates for basal hair cell synapses, and therefore higher metabolic demand and excitotoxicity for higher frequency neurons (Johnson et al., 2008). Alternatively, NR might not adequately reach the apex region. In a different study, IP‐injected NR exerted its benefits on the apex region after noise exposure (Han et al., 2020). Thus, we believe that NR reaches all regions of the cochlea although the administered NR dosage might not be adequate to exert its benefits in the whole cochlea. This potential phenomenon could also explain the lack of mitochondria‐related changes in GO terms or gene expression in Figures 3 and 4 since those studies were done on the whole cochlea, which might mask the effect of NR on cochlear cells in the base region. Alternatively, NR might regulate biological pathways predominately in the base area of the cochlea. For instance, SOD2 protein expression shows a base‐to‐apex increasing gradient in afferent auditory neurons in rodent cochlea (Ying & Balaban, 2009). SOD2 is an antioxidant enzyme inside mitochondria whose levels increase with NR treatment (Cantó et al., 2012). Interestingly, mitochondria expressing more SOD2 show a closer association with lipid droplets (Shiozaki et al., 2011). Regardless, NR's specific impact on high frequencies also suggests that NR's benefit on hearing loss primarily originates from its effect on the cochlea.

Taken together, our study demonstrates the therapeutic potential of NAD+ repletion, using NR, for the treatment of ARHL via improving the synaptic transmission in the cochlea and it points out lipid droplet dynamics as novel NR targets in the cochlea.

## 4 EXPERIMENTAL PROCEDURES

### 4.1 Cell culturing

HEI‐OC1 cells were a generous gift of Dr. Federico Kalinec and their maintenance was previously described (Kalinec et al., 2016). Briefly, we cultured HEI‐OC1 cells in Dulbecco's modified Eagle medium (DMEM) containing 10% fetal bovine serum (FBS) in a humidified chamber under permission conditions (10% CO_2_ at 33°C).

### 4.2 Animals

mtKeima transgenic mice were a generous gift of Dr. Toren Finkel and were described previously (Malide et al., 2016). These mice (http://www.informatics.jax.org/allele/MGI:5660493) are on a mixed genetic background. To rule out the cadherin23 AHL allele as a potential confounder in our analyses, mice were genotyped and found to be all WT for the cadherin23 gene. Additionally, mice were genotyped for the C57BL/6J nicotinamide nucleotide transhydrogenase gene (NNT) mutation and were WT.

NR was given orally in drinking water to mouse strains at a concentration of 7 mg/mL (24 mM), while the non‐treated control groups received regular drinking water. The justification for the NR concentration used in animal studies was described previously (Okur, Mao, et al., 2020). The water bottles, with or without NR, were changed twice per week. 12‐month‐old mtKeima mice were used for quantifying NAD+ levels in the cochlea. NR administration to mtKeima mice started at 2 m and treatment lasted for 10 m until mice were 12 m of age; or started at the age of 15 m and lasted for 1.5 m until 16.5 m of age. The cochlea was dissected for further assessment at the end of the NR administration right after the auditory assessment. The WT CBA/CaJ mice were purchased from The Jackson Laboratory (cat number #000654). NR administration to WT CBA/CaJ mice started at 3 m of age and lasted for 24 m until mice were 27 m of age. Six mice were removed from the cohort for technical reasons such as death during sedation. Thirteen mice in the cohort died due to various reasons before the termination date of 27 months. NR administration displayed no significant effect on the fatality of mice (NR+ [N6, 2F, 4 M] = 23.85 months vs NR− (N7, 3F, 4 M) = 24.6 months, *p* = 0.74). Mice were maintained on a 12 h light–dark cycle and fed ad libitum (Envigo, T.2018SX.15 Global 18% Protein Extruded Rodent Diet [Sterilizable]; https://insights.envigo.com/hubfs/resources/data‐sheets/2018sx‐datasheet‐0915.pdf). All animal protocols were approved by the Animal Care and Use Committee of the Intramural Research Program of the National Institute on Aging, OSD‐361‐2023 in accordance with the National Research Council's Guide for the Care and Use of Laboratory Animals.

### 4.3 NAD+ quantification

Details are provided in the supplementary methods section.

### 4.4 Audiometry

ABR and DPOAE methodology has been described previously (Okur, Mao, et al., 2020). Details are provided in the supplementary methods section.

### 4.5 RNA isolation and quantitative real‐time PCR

The cochlea from treated and non‐treated five male mice (12 m) was dissected. Total RNA from each cochlear tissue was extracted using Trizol Reagent (Zymo Research, #R2050‐1‐50) and Direct‐zol RNA Miniprep (Zymo Research, #R2050) as described previously (Vikhe Patil et al., 2015). Next, one microgram of isolated RNA was reverse transcribed using the iScript™ cDNA Synthesis Kit (BioRad). Using the DyNAmo HS SYBR green qPCR kit (F‐410L, ThermoFisher Scientific) with the CFX connect real‐time PCR detection system (Bio‐Rad), qPCR was performed. Primer sequences are listed in Table S1. Experimental values were normalized to values for GAPDH. The same protocol was followed for RNA isolation from cochlear cells except those steps to break the cochlear bone were skipped.

### 4.6 RNA sequencing

RNA from mtKeima mice cochlea was isolated as described above. Library construction and sequencing were performed by Novogene. Details are provided in the supplementary methods section.

### 4.7 Reagents and immunoblotting

Cochlear tissue and cells were lysed using RIPA buffer (Cell Signaling, #9806) supplemented with Halt™ Protease and Phosphatase Inhibitor Cocktail (Thermo Scientific™, #78444). Western blotting was performed according to the manufacturer's instructions. Briefly, protein concentration was measured using a Pierce™ BCA protein assay kit (Thermo Fisher, 23225). Samples were separated on 4%–12% Bis‐Tris gel (Thermo Fisher Scientific, #NP0336BOX) and transferred to PVDF membranes (BioRad, #1620177). Unless indicated otherwise, membranes were blocked in Tris Buffered Saline (TBS) with 0.1% Tween 20 TBST +3% milk at room temperature for 1H, incubated overnight with primary antibodies, washed 3× with TBST for 5 min, incubated with HRP‐conjugated secondary antibody, and developed using SuperSignal™ West Femto Maximum Sensitivity Substrate (Thermo Scientific™, 34095) according to manufacturer's instructions. Antibodies were used according to the manufacturer's instructions to detect the following antigens: CIDEC/Fsp27 (Ptglab, #12287‐1‐AP), PCK1 (Ptglab, #16754‐1‐AP), Plin1 (Abcam, Ab3526, 1:1000), Sirt1 (Santa Cruz, sc15404, 1:1000), GAPDH (Abclonal, AC027, 1:5000), PPARγ (Abcam, ab59256, 1:1000), CtBP2 IgG1 (BD Biosciences, 612,044, 1:200), GluR2 IgG2a (Millipore, MAB397, 1:1000), myosin VIIa (Novus Biologicals, NB120‐3481, 1:200).

### 4.8 Cochlear immunofluorescence and imaging

Isolated cochlea was punched into oval and round windows using a syringe needle and rinsed/fixed with 4% paraformaldehyde in 1× cold PBS. Then, cochlea was further fixed in 4% paraformaldehyde in 1× cold PBS for an additional 2 h at 4°C. Following fixation, samples were rinsed 3 × 20 min in PBS and dissected under a stereomicroscope to the three turns: apical, middle, and basal. Tissues were permeabilized in PBS + 0.25% Triton X‐100 solution for 10 min at room temperature on a rocking platform; blocked with 10% goat serum and 25 mM glycine for 1 h at RT. Tissues were incubated at 4°C overnight with the following primary antibodies: monoclonal mouse anti‐carboxyl‐terminal binding protein 2 (CtBP2) IgG1 at 1:200 (612,044; BD Biosciences) counterstained with goat anti‐Mouse IgG1 conjugated with Alexa Fluor 568 (#A‐21124), monoclonal mouse anti‐GluR2 IgG2a at 1:1000 (MAB397; Millipore) counterstained with goat anti‐Mouse IgG2a conjugated with Alexa Fluor 488 (#A‐21131), and polyclonal rabbit anti‐myosin VIIa at 1:200 (NB120‐3481; Novus Biologicals) counterstained with goat anti‐Rabbit IgG (H + L)conjugated with Alexa Fluor 647 (#A‐21244). Antibodies were added with 1% goat serum. The following day, after three 15‐min PBS washes, the tissues were incubated with the Alexa‐conjugated secondary antibodies at a concentration of 1:600 for 1 h in darkness at room temperature. Following the final washes after secondary incubations, samples were carefully mounted on slides using ProLong Glass antifade media and left to dry for at least 24 h before image acquisition. Frequency regions corresponding to 16 and 32 kHz were located through their distance from cochlear apex, based on the place‐frequency map from Müller et al. (2005) and imaged with a 63× 1.4NA Plan Apo objective on a Zeiss 880 LSM Airyscan confocal microscope with a 47 nm xy pixel size (Carl Zeiss). After acquisition, images were Airyscan processed.

### 4.9 Ex‐vivo mitophagy analysis in the cochlea

Ex‐vivo mitophagy analysis in mtKeima mice has been described previously (Malide et al., 2016). The fluoresce reporter contains a coral‐derived Keima protein that is targeted to mitochondria using a mitochondria‐targeting sequence from COX VIII. Mt‐Keima protein exhibits pH‐dependent two excitation maxima: near 488 nm at pH 8 and near 561 at pH 4, allowing to distinguish free mitochondria (pH 8) from mitochondria in mito‐lysosomes (pH 4). Details are provided in the supplementary methods section.

### 4.10 Statistical analysis

Two‐tailed *t*‐test was used to determine the differences between the two groups while One‐way ANOVA with Tukey's post hoc test was used to determine significant differences across multiple samples unless indicated otherwise in figure panels. Mixed effect analysis with Sidak's multiple comparison test was used to determine significant differences in ABR thresholds in NR‐treated and non‐treated groups. Statistical analyses were performed with GraphPad Prism version 7 (GraphPad Software, Inc.).

## AUTHOR CONTRIBUTIONS

Mustafa N. Okur, Vilhelm A. Bohr, and Deborah L. Croteau planned the study. Mustafa N. Okur, Risako Kimura, Jae‐Hyeon Park, Burcin Duan Sahbaz, Chandrakala Puligilla, Uri Manor, and Leo Andrade conducted the experiments. Mustafa N. Okur, Uri Manor, and Leo Andrade contributed to image acquisition, processing, and analysis. Deborah L. Croteau and Mustafa N. Okur conducted gene expression analysis. Mustafa N. Okur wrote the manuscript. Mustafa N. Okur, Vilhelm A. Bohr, Deborah L. Croteau, and Uri Manor edited the manuscript and contributed to the interpretation of the results.

## CONFLICT OF INTEREST STATEMENT

Dr. Vilhelm Bohr previously had a CRADA agreement with ChromaDex.

## Supporting information


Figure S1.
Figure S2.
Figure S3.
Figure S4.
Figure S5.
Figure S6.
Figure S7.
Figure S8.

Table S1.

## Data Availability

RNA sequencing data were submitted Gene Expression Omnibus (GEO). The GEO accession code is GSE213994.
