# Supplementary material for: Long‐term NAD+ supplementation prevents the progression of age‐related hearing loss in mice
Source: Aging Cell. 2023 Jul 3;22(9):e13909. doi: 10.1111/acel.13909 (PMC10497810; doi:10.1111/acel.13909)
Supplement: Supplementary file 1 — Figure S1. Figure S2. Figure S3. Figure S4. Figure S5. Figure S6. Figure S7. Figure S8. [file ACEL-22-e13909-s001.pdf]

Supplementary Tables and Figures

Long-term NAD+ supplementation prevents the progression of age-related hearing loss in mice

Supplementary Figure 1

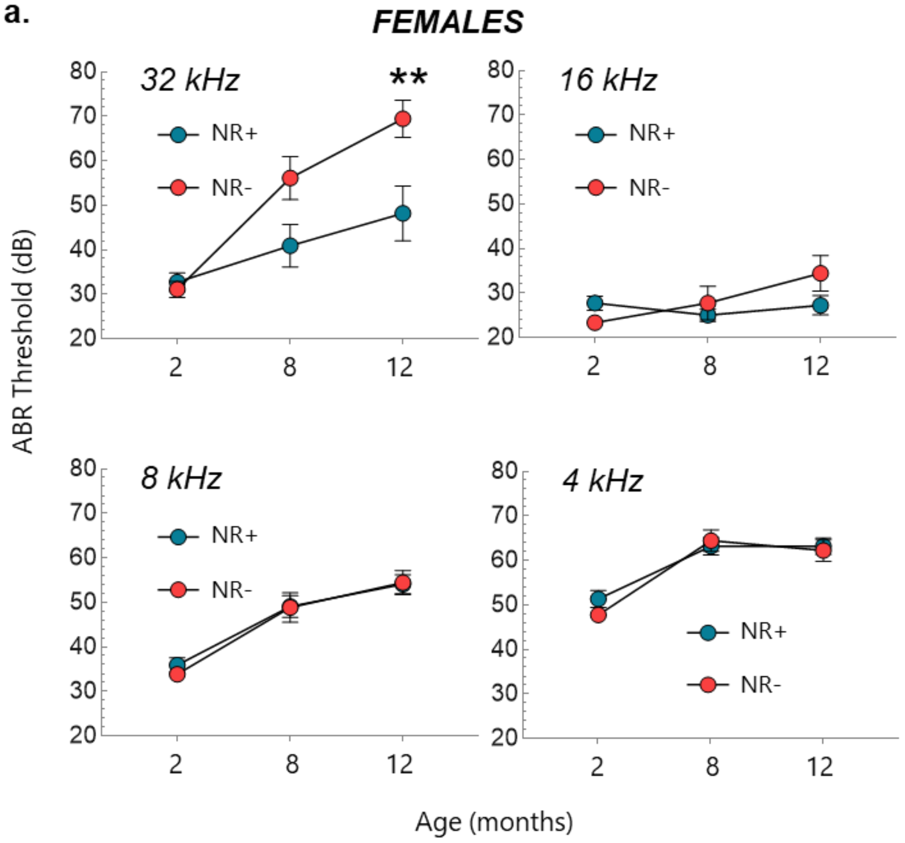

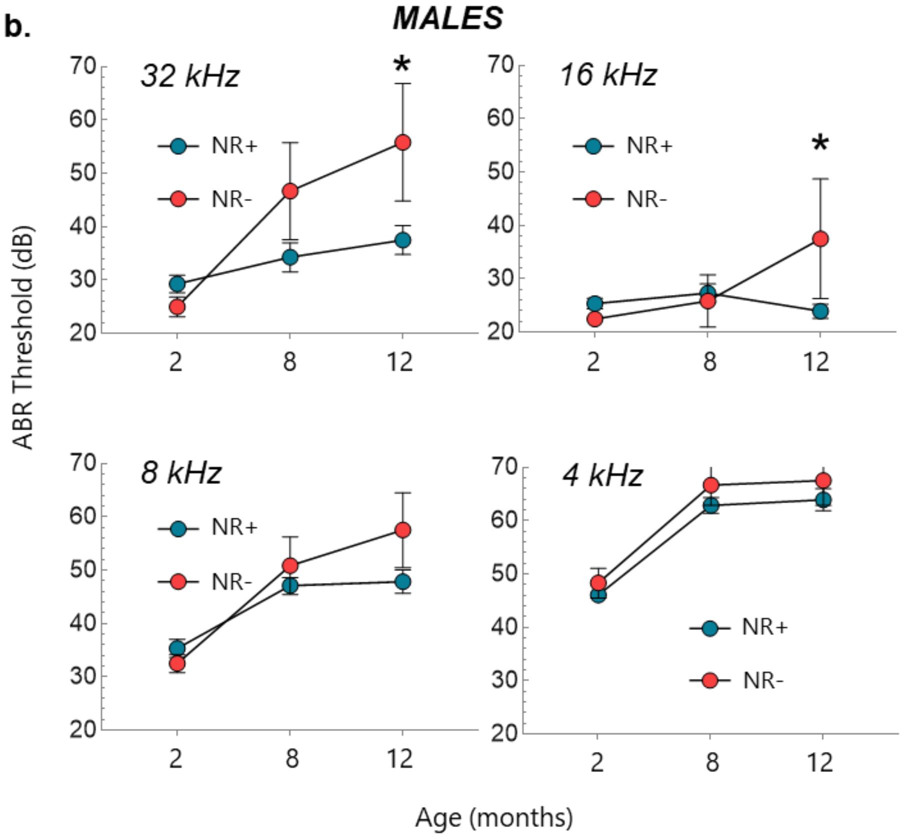

Supplementary Figure 2

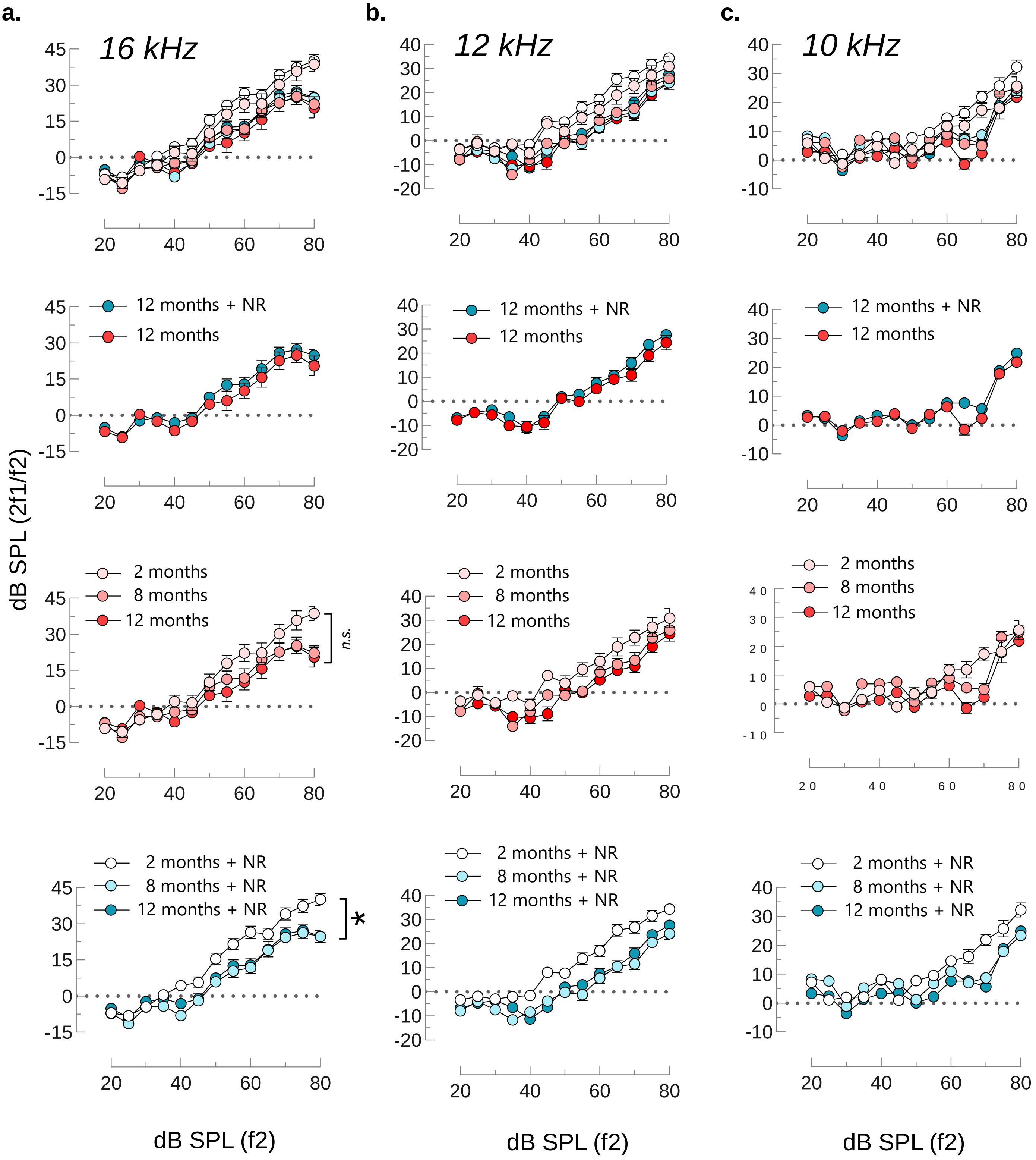

Supplementary Figure 3

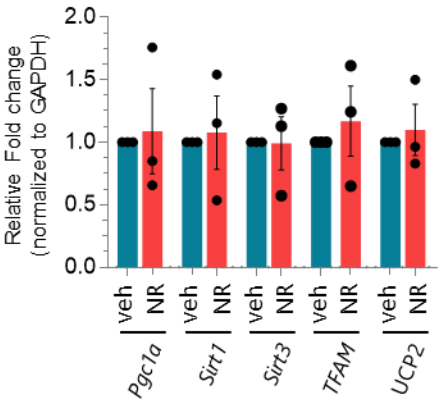

Supplementary Figure 4

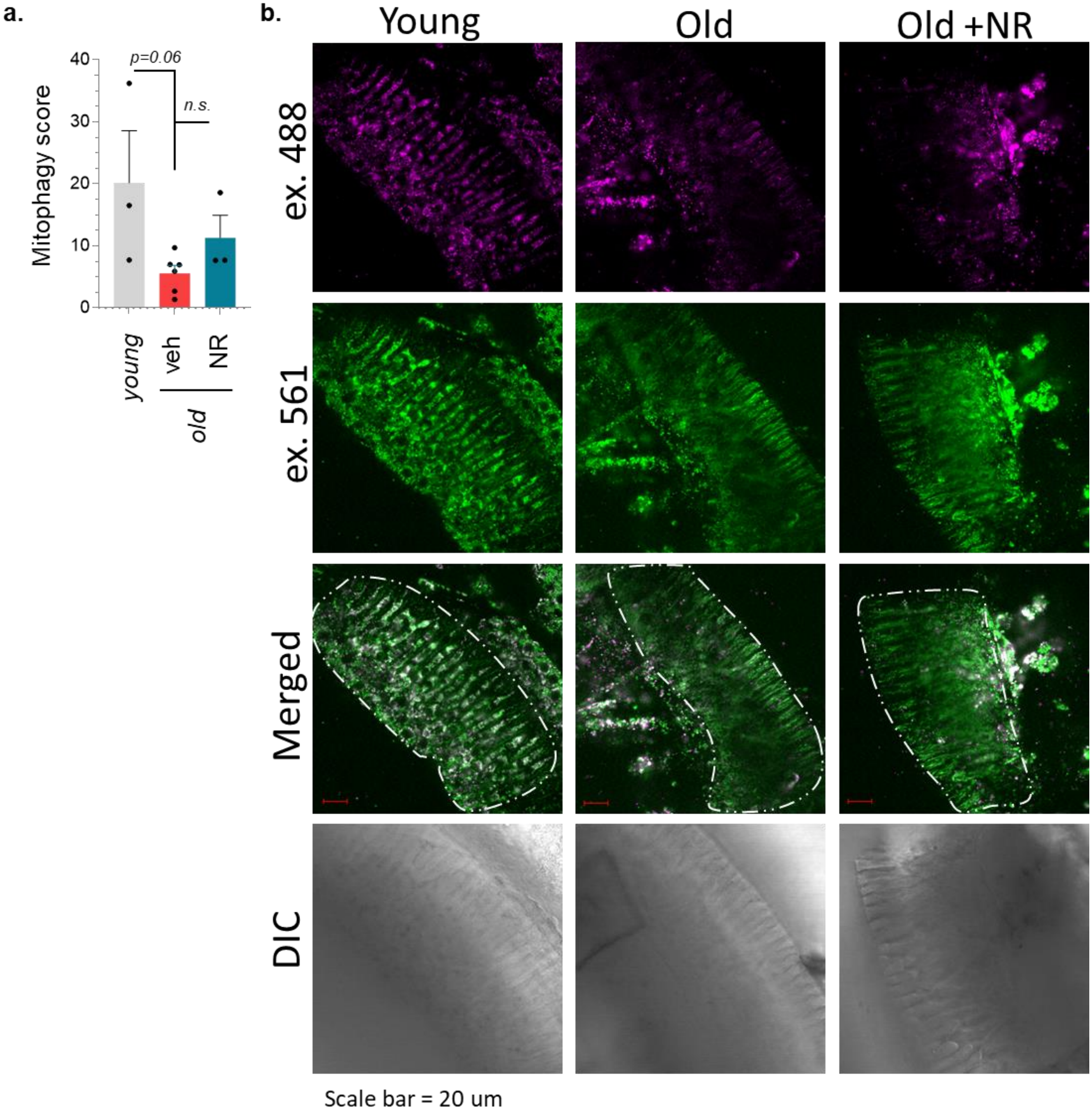

Supplementary Figure 5

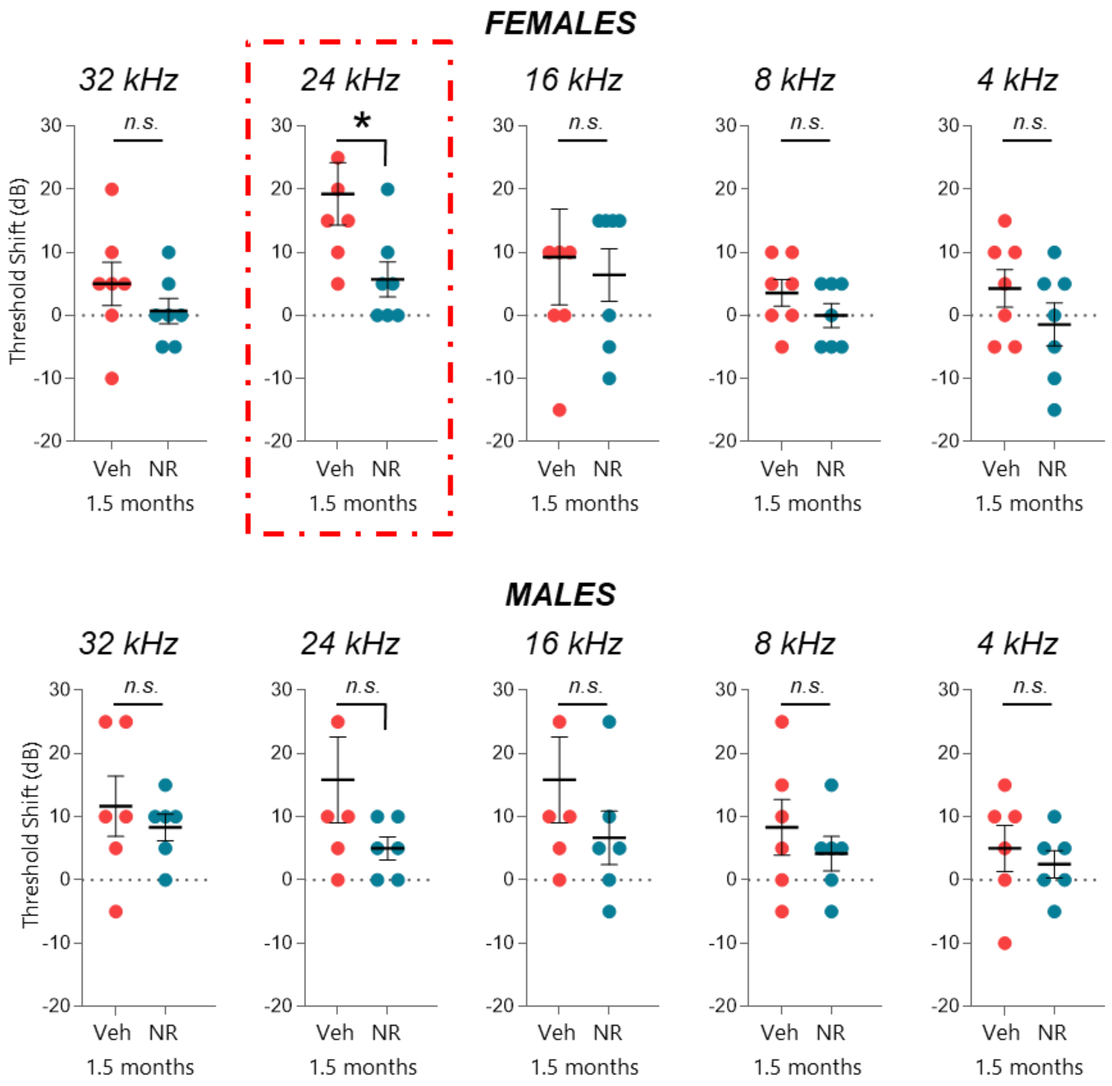

Supplementary Figure 6

a.

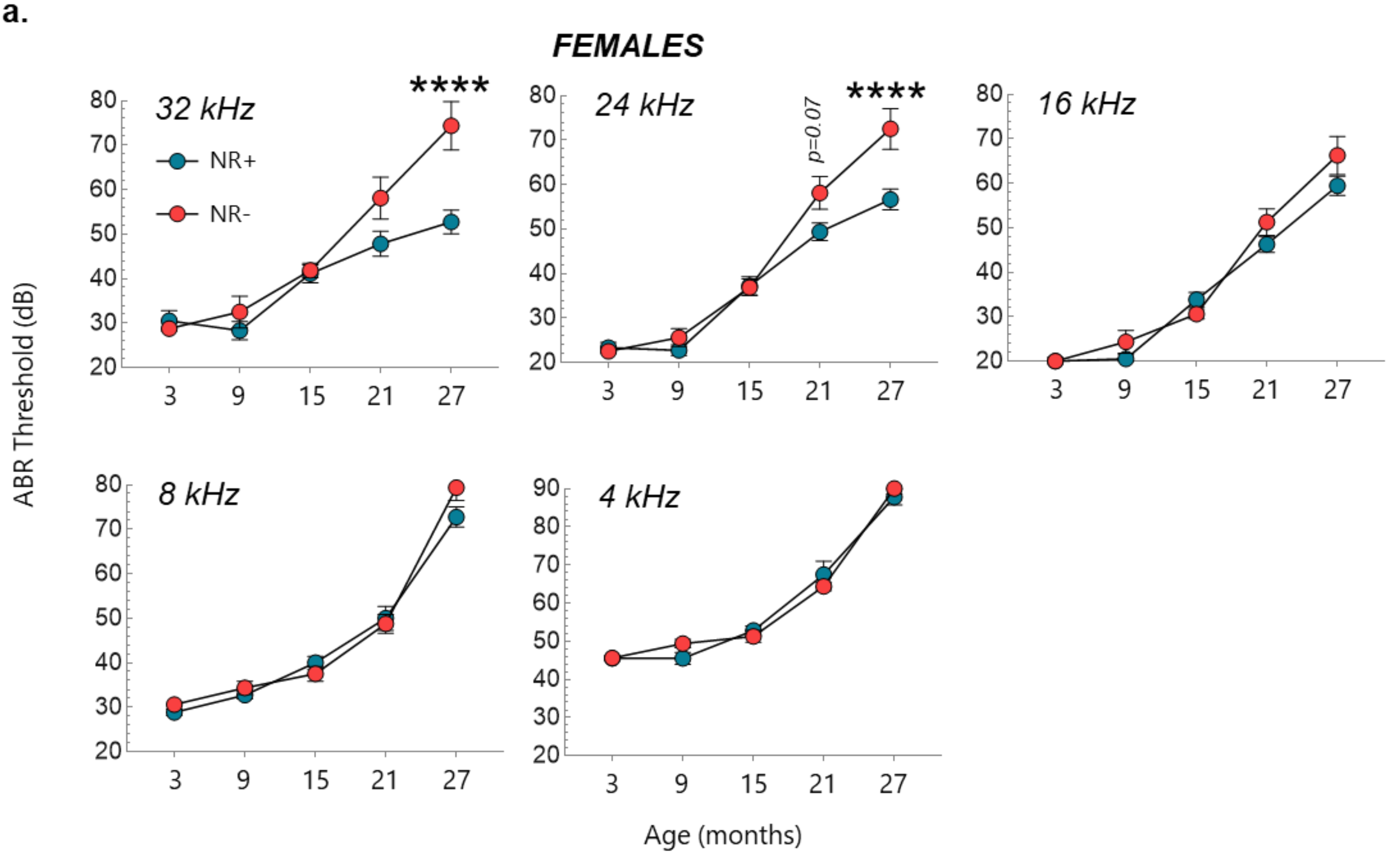

b.

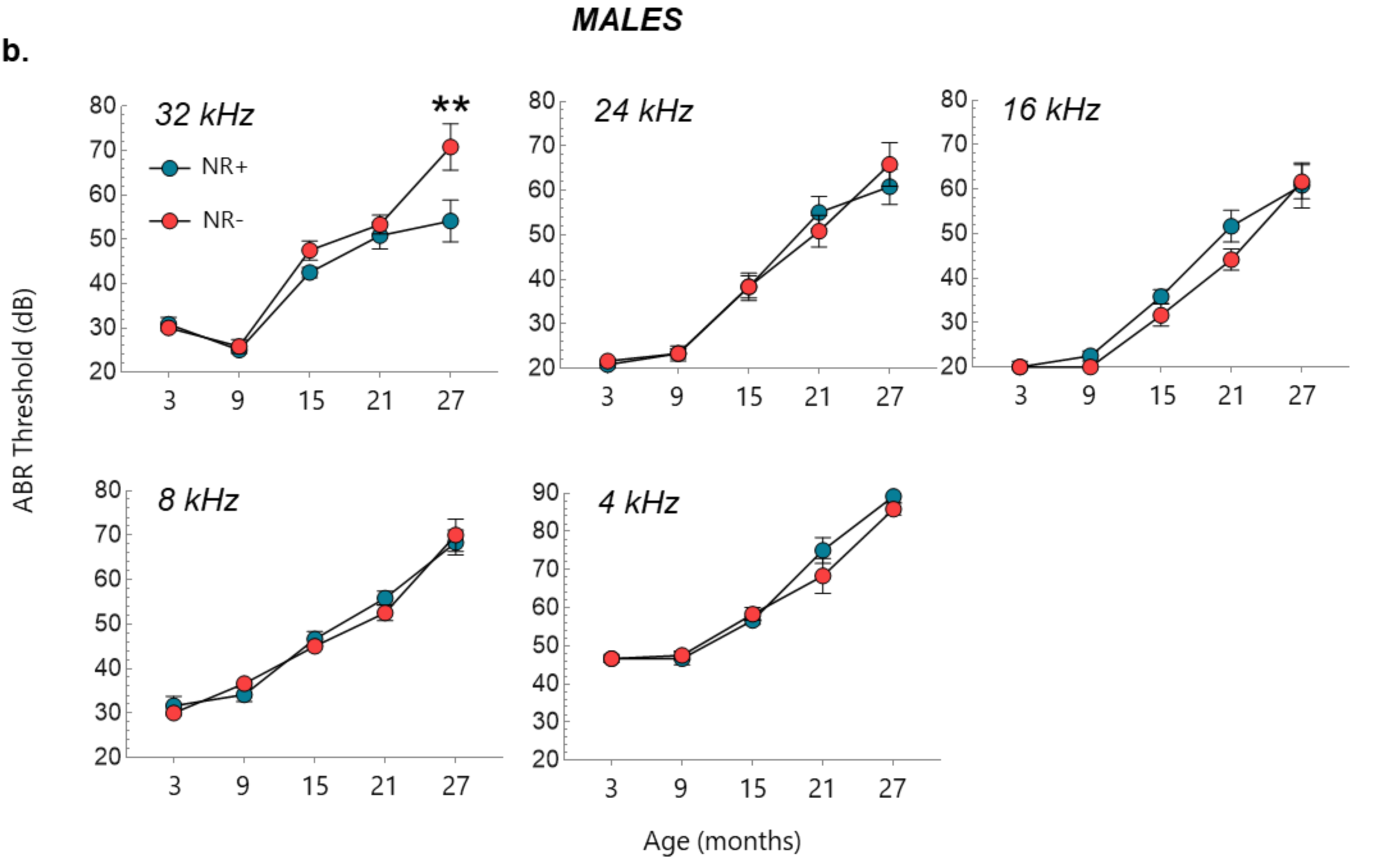

Supplementary Figure 7

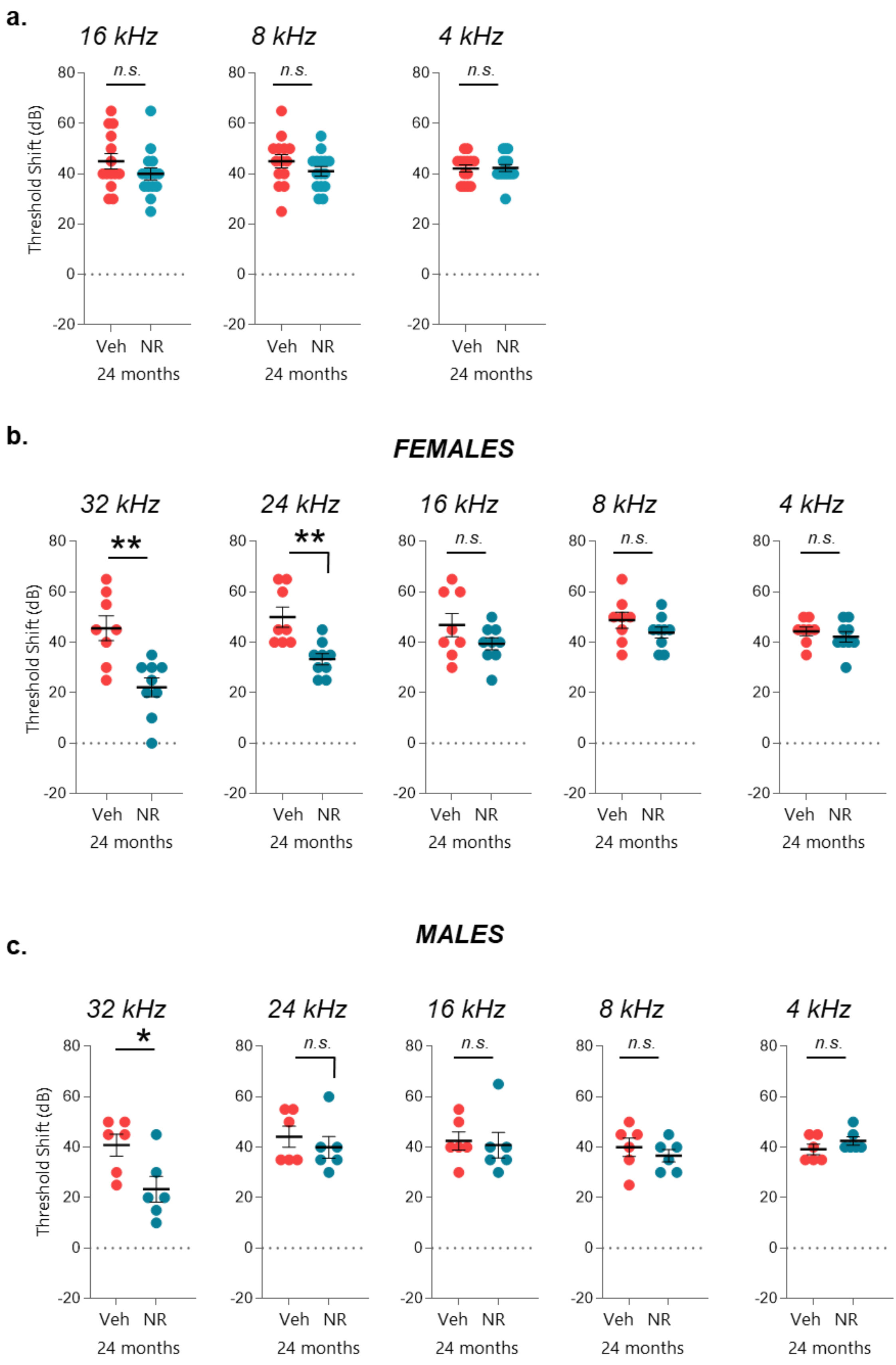

Supplementary Figure 8

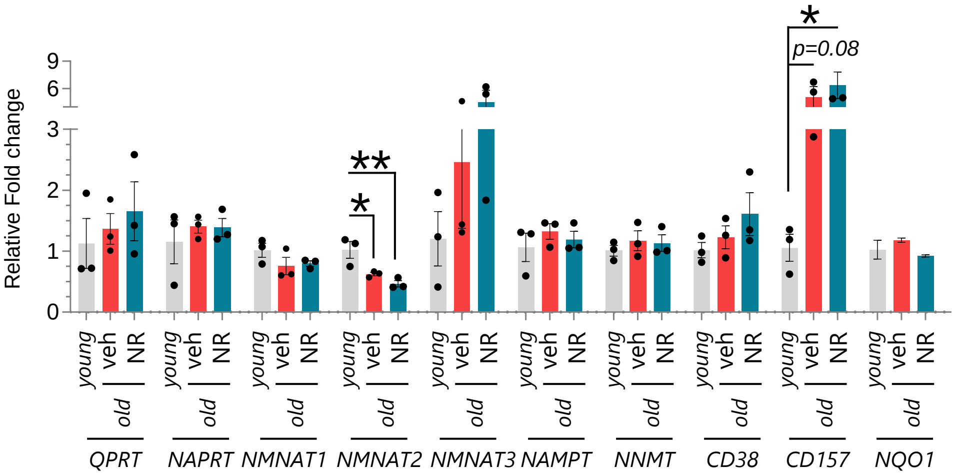

Supplementary Table S1

	sense	antisense
PCK1	ATGAAAGGCCGCACCATGTA	GCACAGATATGCCCATCCGA
PPARγ	TGT GGG GAT AAA GCA TCA GGC	CCG GCA GTT AAG ATC ACA CCT AT
CIDE-C (cidec)	ATG GAC TAC GCC ATG AAG TCT	CGG TGC TAA CAC GAC AGG G
Plin1	GTG CTT CCA GAA GAC CTA CAA	CTT CAG TTC AGA GGC GAT CTT
Pgc-1α	GGA CCA GAT GCG TTC TCT ATT T	CTA CCC ACA GTG TCT GCA TAA G
Sirt1	CAG TGT CAT GGT TCC TTT GC	CAC CGA GGA ACT ACC TGA T
Sirt3	TACAGGCCCAATGTCACTCA	ACAGACCGTGCATGTAGCTG
Tfam	CCA AAA AGA CCT CGT TCA GC	ATG TCT CCG GAT CGT TTC AC
Sod1	AAC CAG TTG TGT TGT CAG GAC	CCA CCA TGT TTC TTA GAG TGA GG
Gpx1	CCA CCG TGT ATG CCT TCT CC	AGA GAG ACG CGA CAT TCT CAA T
Cat	GGA GGC GGG AAC CCA ATA G	GTG TGC CAT CTC GTC AGT GAA
Ucp2	ATG GTT GGT TTC AAG GCC ACA	TTG GCG GTA TCC AGA GGG AA
NMNAT1	GAG AAC TGA CAC GTC CTT AGT G	CCT TGA GCC TTT GAT CCT TCT
NMNAT2	TGA TGC TCT CCT GTG GTT TAC	ACC CTA CTC TTC CCA GAC TTA T
NMNAT3	CTC TGG GTT CTG GGT TTG TT	CCC TGT CAA ACT CTG CCT ATC
QPRT	CGG TTC TAA AGC CGA AGA AGA	ATC ATC AAC AGC CCA GTA AGG
NAPRT	GTC TTT GCT ACT GGA CCT ACT G	GGG AAC AGA GCC GTG TTA TT
NAMPT	CAC TTC GGT GGC AGA AAT AGA	GTC TTA GGA TCA CCT GCT AAC C
NNMT	CCA CCA TCT ATC AGC TTC TCT C	CTC TGG GTC ACA TCA CAC TTT A
CD38	TCT CTC TCT CTC TCT CTC TCT CT	CAC ACA GCA CCT TCC CTA TAA T
CD157	CAC GGG CTA GAG GAA TCA AAG	GCC TGG AGA GGT TAA TGA AGA G
NQO1	GAG AAG AGC CCT GAT TGT ACT G	GCG TCC TTC CTT ATA TGC TAG AG
Gapdh	Predesigned qPCR from IDTDNA were used. Cat # Mm.PT.39a.1	
