# Supplementary material for: Long‐term NAD+ supplementation prevents the progression of age‐related hearing loss in mice
Source: Aging Cell. 2023 Jul 3;22(9):e13909. doi: 10.1111/acel.13909 (PMC10497810; doi:10.1111/acel.13909)
Supplement: Supplementary file 2 — Table S1. [file ACEL-22-e13909-s002.docx]

**Long-term NAD+ supplementation prevents the progression of age-related hearing loss in mice**

**SUPPLEMENTARY METHODS**

***Audiometry (continued)***

Briefly, mice were anesthetized with ketamine (100 mg/kg) and xylazine (10 mg/kg) via an intraperitoneal (i.p.) injection and placed in a soundproof chamber on a heating pad in such a way that the recorded ear was 7 cm away from the sound source (MF1 Multi-Field Magnetic Speaker). After inserting the needle electrodes sub-dermally (vertex–ventrolateral to pinna), tone burst stimuli (5 ms duration with a 0.1-ms rise-fall time) were presented at variable volume (10–90 dB SPL) in 5 dB steps at 4, 8, 16, and 32 kHz using RZ6 system (Tucker Davis Technologies) with Biosig software (Tucker Davis Technologies). The minimum volume threshold (in dB) that evokes a response at a given frequency was recorded as the outcome measure. The waveforms were determined by an average of 512 responses. The ABR threshold was determined by visual inspection and considered to be the lowest stimulus level at which at least one wave was present.

Raw ABR recording data were extracted using the BioSigRZ software. Voltage values were sampled at a rate of 50,000 Hz (every 0.02 ms) for a duration of 4.5 ms following stimulus presentation. Representative waveforms were calculated and reconstructed offline by averaging the voltage values at each time point using TDT BioSigRZ software. Waves I-V were identified by a series of characteristic peak-to-following-trough forms.

DPOAE measurement was described previously ^9^. Briefly, an earplug connected to a small microphone (ER-10B+) and two speakers (MF1 Multi-Field Magnetic Speaker) was inserted into the outer ear canal of each mouse. Using the RZ6 system (Tucker Davis Technologies) with Biosig software (Tucker Davis Technologies), a series of auditory stimuli were delivered to the speaker, each composed of two tones at equal decibel levels but distinct frequencies, *f*1 and *f*2, where *f*2 > *f*1, *f*2/*f*1 = 1.2 at *f*0 = 10, 12, 16, and 32 kHz (*f*0 = (*f*1 × *f*2)1/2). The decibel level of both tones varied over the range of 80 dB SPL to 10 dB SPL in 5-dB steps. The distortion product at the frequency 2*f*1 − *f*2 was recorded at each frequency tested as an average of 512 responses.

***NAD+ quantification (continued)***

Dissected cochlea were placed in NADH/NAD extraction buffer (Abcam, ab65348) and homogenized with a micro pestle. NAD+ and NADH levels in cochlea were quantified using the NAD/NADH Assay Kit per manufacturer instructions. Samples were normalized to total protein concentration in each cochlea using Pierce™ BCA Protein Assay Kit. Note: NAD+ samples in CBA/CaJ cochlear samples were measured 1.5 years after their extraction from the mice.

***RNA sequencing (continued)***

Library construction and sequencing were performed by Novogene. RNA purity was checked using the NanoPhotometer® spectrophotometer (IMPLEN, CA, USA). RNA integrity and quantitation were assessed using the RNA Nano 6000 Assay Kit of the Bioanalyzer 2100 system (Agilent Technologies, CA, USA). A total amount of 1 μg RNA per sample was used as input material for the RNA sample preparations. Sequencing libraries were generated using NEBNext® UltraTM RNA Library Prep Kit for Illumina® (NEB, USA) following the manufacturer’s recommendations and index codes were added to attribute sequences to each sample. Briefly, mRNA was purified from total RNA using poly-T oligo-attached magnetic beads. Fragmentation was carried out using divalent cations under elevated temperature in NEBNext First Strand Synthesis Reaction Buffer (5X). First-strand cDNA was synthesized using random hexamer primers and M-MuLV Reverse Transcriptase (RNase H-). Second strand cDNA synthesis was subsequently performed using DNA Polymerase I and RNase H. Remaining overhangs were converted into blunt ends via exonuclease/polymerase activities. After adenylation of 3’ ends of DNA fragments, the NEBNext adaptor with a hairpin loop structure was ligated to prepare for hybridization. To select cDNA fragments preferentially of 150~200 bp in length, the library fragments were purified with the AMPure XP system (Beckman Coulter, Beverly, USA). Then 3 μl USER™ Enzyme (NEB, USA) was used with size-selected, adaptor-ligated cDNA at 37 °C for 15 min followed by 5 min at 95 °C before PCR. Then PCR was performed with Phusion High-Fidelity DNA polymerase, Universal PCR primers, and Index (X) Primer. At last, PCR products were purified (AMPure XP system) and library quality was assessed on the Agilent Bioanalyzer 2100 system. The clustering of the index-coded samples was performed on an Illumina Novaseq sequencer according to the manufacturer’s instructions. After cluster generation, the libraries were sequenced on the same machine and paired-end reads were generated.

RNAseq analysis was provided by Novogene. Original image data file from Illumina was transformed to sequenced reads (raw data) by CASAVA base recognition (base calling). Datasets are being uploaded to GEO. Raw data was subjected to data QC including error rate distribution, GC content distribution, and data filtering. Mapping was performed using STAR (v2.6.1d, mismatch=2) and reads were mapped to the mouse reference genome mm10. Quantification was conducted by FeatureCounts (v1.5.0) under default mode. Normalization and differential gene expression used DESeq2 (v1.26.0) ^45^. Genes with p-value ≤0.05 were passed on for enrichment analysis (Gene Ontology and KEGG) using ClusterProfiler (v3.8.1) and terms with padj <0.05 were considered significant.

***Ex-vivo mitophagy analysis in the cochlea (continued)***

Ex-vivo mitophagy analysis on cochlear tissues was performed as follows: Dissected temporal bone is placed in a silicone elastomer-coated dissection dish filled with 1X PBS at 4°C. Cochlea with otic capsule was removed from temporal bone and stabilized on the dish using pins. The otic capsule was slowly snipped off using Dumont #5 Fine Forceps (Fine Science Tools, #11254-20) and Vannas-Tübingen Spring Scissors (Fine Science Tools, #15003-08) and separated from cochlea inside the capsule without damaging the cochlear structure. The cochlea is then fragmented into the apex, middle, and base and placed on Nunc™ Glass Bottom Dishes (Thermo Scientific™, #150680) with 1X PBS at 4°C containing DAPI (Thermo Scientific™, #62248) at a final concentration of 1/3000 mg/ml. Following 10 mins of incubation, excess PBS was removed from the plate, and cochlea were imaged using a Zeiss 880 LSM confocal microscope.
